# Placental Glucose Transporters and Response to Bisphenol A in Pregnancies from of Normal and Overweight Mothers

**DOI:** 10.3390/ijms22126625

**Published:** 2021-06-21

**Authors:** Leonardo Ermini, Anna Maria Nuzzo, Francesca Ietta, Roberta Romagnoli, Laura Moretti, Bianca Masturzo, Luana Paulesu, Alessandro Rolfo

**Affiliations:** 1 Department of Life Sciences, University of Siena, 53100 Siena, Italy; leonardo.ermini@unisi.it (L.E.); roberta.romagnoli@unisi.it (R.R.); luana.riccipaulesu@unisi.it (L.P.); 2 Department of Surgical Sciences, University of Turin, Via Ventimiglia 3, 10126 Turin, Italy; a.nuzzo@unito.it (A.M.N.); l.moretti@unito.it (L.M.); alessandro.rolfo@unito.it (A.R.); 3 Città della Salute e della Scienza, Sant’Anna University Hospital, University of Turin, 10126 Turin, Italy; bmasturzo@cittadellasalute.to.it

**Keywords:** BPA, GLUT1, GLUT4, human pregnancy, human placenta

## Abstract

Bisphenol A (BPA) is a synthetic phenol extensively used in the manufacture of polycarbonate plastics and epoxy resins and a component of liquid and food storages. Among health disorders potentially attributed to BPA, the effects on metabolism have been especially studied. BPA represents a hazard in prenatal life because of its presence in tissues and fluids during pregnancy. Our recent study in rats fed with BPA showed a placental increase in glucose type 1 transporter (GLUT-1), suggesting a higher uptake of glucose. However, the role of BPA on GLUT transporters in pregnant women with metabolic dysfunction has not yet been investigated. In this study, placental tissue from 26 overweight (OW) women and 32 age-matched normal weight (NW) pregnant women were examined for expression of GLUT1 and GLUT4. Placental explants from OW and NW mothers were exposed to BPA 1 nM and 1 μM and tested for GLUTs expression. The data showed a different response of placental explants to BPA in GLUT1 expression with an increase in NW mothers and a decrease in OW ones. GLUT4 expression was lower in the explants from OW than NW mothers, while no difference was showed between OW and NW in placental biopsies for any of the transporters.

## 1. Introduction

Bisphenol A (BPA) is a synthetic phenol extensively used in the manufacture of polycarbonate plastics and epoxy resins and a component of many everyday products such as liquid and food storages (water plastic bottles, beverage can linings, and food packaging), detergents, and medical and dental devices. Consequently, the human population is continuously exposed to BPA, as revealed by its presence in the environment and populations in general [1,2,3,4]. BPA can represent a hazard in prenatal life because of its presence in the placenta, amniotic fluid, maternal and fetal blood, and its ability to cross the placenta and reach the fetus [5]. Among health disorders potentially attributed to BPA, its effects on the metabolism have been largely documented in the last few years. Both in human and animal studies a positive association has been found between exposure to a low dose of BPA and energy balance alteration, a predisposition for type 2 diabetes and metabolic disorders (i.e., decreased glucose tolerance and increased insulin resistance) [6]. Remarkably, the same developmental exposure reduces adipocyte number but increases adipocyte volume, with a rise in body weight [7]. Indeed, BPA is a potential “obesogen”, and human exposure to this chemical, particularly during prenatal life, is thought to be a major contributing factor to obesity [8].

Obesity is a global health concern that has increased in the last decades [9]. Obesity in women during their reproductive age or during pregnancy has been associated with an impairment in fertility and fecundity as well as a higher incidence of miscarriage and pregnancy pathologies including gestational hypertension, preeclampsia, nephropathy, retinopathy, and gestational diabetes [10,11,12]. Maternal obesity is also associated with an increased risk in delivering hypoglycaemic, Large for Gestational Age (LGA) or macrosomic infants [10,13].

However, the role of maternal obesity in BPA response during pregnancy has not been elucidated. In this study, we examined healthy pregnant women with normal Body Mass Index (BMI) (normal weight) (CTRL) and women with high BMI (overweight) (OW). Placental tissues after term delivery were examined for expression of glucose transporters (GLUT) 1 and 4 and for the in vitro response to BPA.

This study was based on our previous data in rats fed with a diet containing BPA for a period of a month (virgin state) plus 20 days during pregnancy. The study showed changes in fetal and placental weight leading to higher fetal growth and an increased placental expression of GLUT-1. Upregulation of GLUT1 expression and glucose transfer was also shown in human trophoblast cells [14]. Glucose is one of the main nutrients for fetal development. It is essential for fetal growth and metabolism as the fetus is not capable of performing gluconeogenesis. Therefore, fetal glucose is critically dependent on the placental ability to extract glucose from maternal blood and to transport it to fetal tissues by means of facilitative transport proteins, called GLUT. Among the seven isoforms of glucose transporters in the human placenta, GLUT1, 3, 4, 8, 9, 10, and 12, GLUT1 and 4, have a key role in the regulation of glucose transport [15].

Based on the obesogenic effect of BPA and its impact on GLUT1 placental transport, we hypothesized that maternal metabolic dysfunction and exposure to BPA in a pregnant woman during human pregnancy could interfere with placental glucose transport via GLUTs modulation.

## 2. Results

### 2.1. Clinical Features of Study Population

The clinical features of the studied population are reported in Table 1, Table 2 and Table 3. CTRL (*n* = 32) and OW (*n* = 26) pregnancies were comparable for maternal and gestational age at delivery. As expected, pre-pregnancy (*p* < 0.001) and delivery BMI (*p* < 0.001), plicometry (*p* < 0.001) and mid-upper arm circumference (*p* < 0.001) resulted significantly increased in OW compared to the CTRL pregnancies. In contrast, gestational weight increase was significantly reduced in OW compared to control patients (*p* < 0.001). The descriptive results of the pre-pregnancy BMI categories indicated that 51.8% and 48.2% of participants started pregnancy in an overweight and obese category, respectively. Before 14 weeks of gestation, triglycerides levels were significantly increased in OW compared to CTRL (*p* = 0.018), while during the third trimester, OW patients presented increased glycemia (*p* = 0.006) and systolic blood pressure values (*p* = 0.012) compared to the controls. Gestational Diabetes Mellitus (GDM) was more frequent in OW (37%) compared to CTRL (12.5%) (*p* = 0.027). Importantly, all GDM patients were treated with diet therapy. Even though no significant differences in birth and placental weights were reported between groups (*p* > 0.05), a significantly increased percentage of LGA fetuses was described in OW vs. CTRL (*p* = 0.024).

### 2.2. Placental GLUT1 and GLUT4 Expression in Normal and Overweight Pregnancies

**Figure 1:**
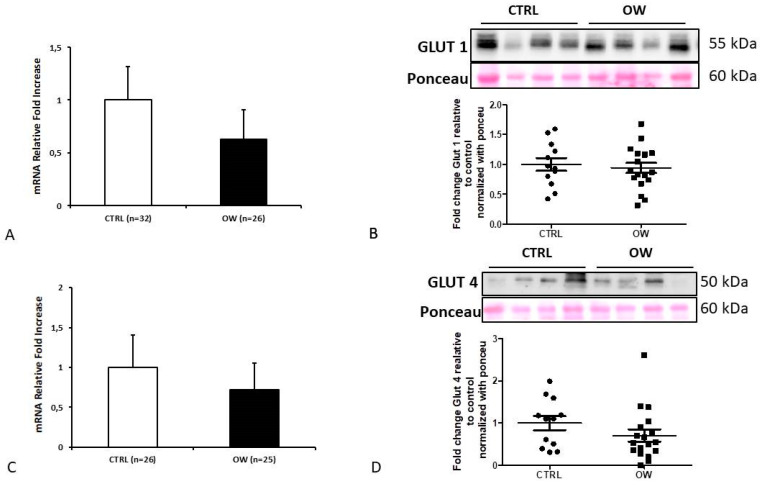
Glut 1 and 4 expression (**A**,**C**) and protein levels (**B**,**D**) in placenta from normal (**CTRL**) and overweight (**OW**) women. (**A**) qPCR analysis of GLUT1 mRNA in normal (*n* = 32) and overweight (*n* = 26) placental tissue. (**B**) Representative WB (high panel) and corresponding densitometry (low panel) of GLUT1 in placenta from CTRL (*n* = 12) and OW (*n* = 18) women. (**C**) qPCR analysis of GLUT4 mRNA in normal (*n* = 26) and overweight (*n* = 25) placental tissue. (**D**) Representative WB (high panel) and corresponding densitometry (low panel) of GLUT4 in placenta from CTRL (*n* = 12) and OW (*n* = 18) women. Data are presented as mean ±ES.

qPCR and Western blot analysis were used to examine if the high BMI levels of women could affect placental glucose transporter mRNA and protein expression. We reported no significant differences in GLUT1 (*p* > 0.05, 1.61-Fold Decrease) and GLUT4 (*p* > 0.05, 1.38 Fold Decrease) mRNA levels in placentae obtained from normal (NW) and overweight (OW) women (Figure 1a,c). These data were confirmed at protein levels where a Western blot analysis for GLUT1 (*p* > 0.05; 0.9403-Fold Change) and GLUT4 (*p* > 0.05; 0.7066 Fold Change) did not reveal a significant difference between NW and OW placentae (Figure 1b,d).

### 2.3. GLUT1 and GLUT4 Expression in Cultured Placenta Explants from Normal Weight and Overweight Pregnancies

**Figure 2:**
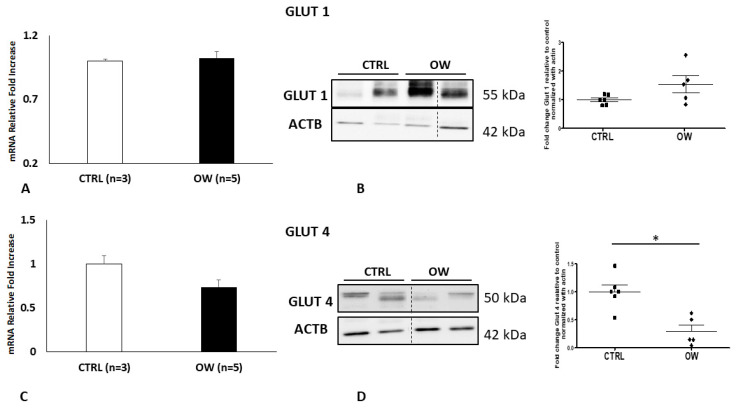
GLUT 1 and 4 expression (**A**,**C**) and protein levels (**B**,**D**) in placenta explants from normal (**CTRL**) and overweight (**OW**) women. (**A**) Fold Change of GLUT1 mRNA in normal (*n* = 3) and overweight (*n* = 5) placental explants. (**B**) Representative WB (left panel) and corresponding densitometry (right panel) of GLUT1 in placenta explants from CTRL (*n* = 6) and OW (*n* = 5) women. (**C**) Fold Change of GLUT4 mRNA in normal (*n* = 3) and overweight (*n* = 5) placental explants. (**D**) Representative WB (left panel) and corresponding densitometry (right panel) of GLUT4 in placenta explants from CTRL (*n* = 6) and OW (*n* = 5) women. Data are presented as mean ±ES. Significance was determined using an unpaired two-sided *t*-test. * *p* < 0.05.

To focus on the expression of glucose transporters in the chorionic epithelium from NW and OW women, we cultured chorionic villous explants for 48 h in a medium containing a physiological concentration of glucose. qPCR and Western blot analysis for GLUT1 (Figure 2a,b) showed no significant variation between NW and OW women, although an increasing trend was observed in the protein levels (*p* = 0.0872; Fold Change 1.537).

On the other hand, quantitative PCR showed a reduction in GLUT4 expression (*p* = 0.0847; Fold Change 0.7383) (Figure 2c). Moreover, the transporter protein levels were significantly decreased (*p* = 0.0024; Fold Change 0.2910) in placenta explants obtained by the women with high BMI compared to the normal ones (Figure 2c,d).

### 2.4. BPA Impact in Placenta Explants from Normal Weight Pregnancies

**Figure 3:**
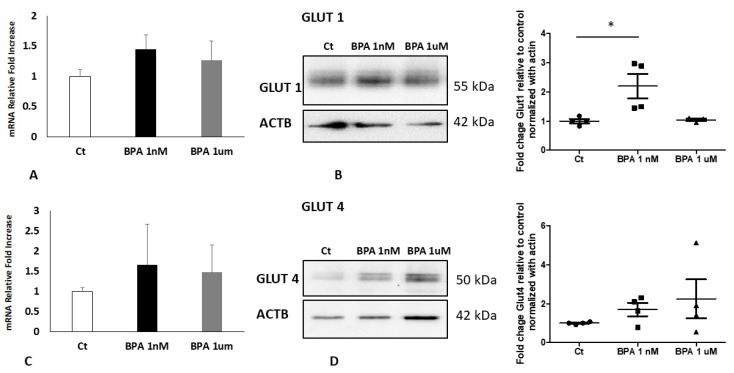
GLUT 1 and 4 expressions (**A**,**C**) and protein levels (**B**,**D**) in placenta explants treated with BPA from control pregnancy. (**A**) Fold Change of GLUT1 mRNA in placental explants from NW women (*n* = 3) treated with BPA 1 nM and BPA 1 μM compared to the vehicle as control. (**B**) Representative WB (left panel) and corresponding densitometry (right panel) of GLUT1 in placental explants from NW women (*n* = 4) treated with BPA 1 nM and BPA 1 μM compared to the vehicle as control. (**C**) Fold Change of GLUT4 mRNA in placental explants from NW women (*n* = 3) treated with BPA 1 nM and BPA 1 μM compared to the vehicle as control. (**D**) Representative WB (left panel) and corresponding densitometry (right panel) of GLUT4 in placental explants from NW women (*n* = 4) treated with BPA 1 nM and BPA 1 μM compared to the vehicle as control. Data are presented as mean ±ES. Significance was determined using a one-way ANOVA and Bonferroni’s test for post hoc comparisons * *p* < 0.05. Ct: control.

Studies carried out by Benincasa et al. showed an increase in GLUT1 levels in a human trophoblast cell line treated with BPA [14]. To explore if the endocrine disruptor could have the same effect on our in vitro model, the placental explants from physiological pregnancies of NW women were treated with BPA at two different concentrations (1 nm and 1 μm) or vehicle as control (Ct). The concentrations used are considered physiologically relevant as routinely detected in the blood and other body fluids of the general population [1,16]. The explants were then subjected to RNA and protein extraction followed by qPCR and a WB analysis for the transporters GLUT1 and GLUT4 as described in the material and method section. In line with the findings in the trophoblast cells [14], GLUT1 protein expression was significantly increased (*p* < 0.05; Fold Change 2.200) in placenta explants by BPA treatment. Similarly, the effect was obtained at 1 nM, while 1 μM was ineffective. No significant changes were observed in GLUT1 mRNA nor in GLUT4 protein and gene expression (Figure 3).

### 2.5. BPA Impact in Placenta Explants from Overweight Pregnancies

**Figure 4:**
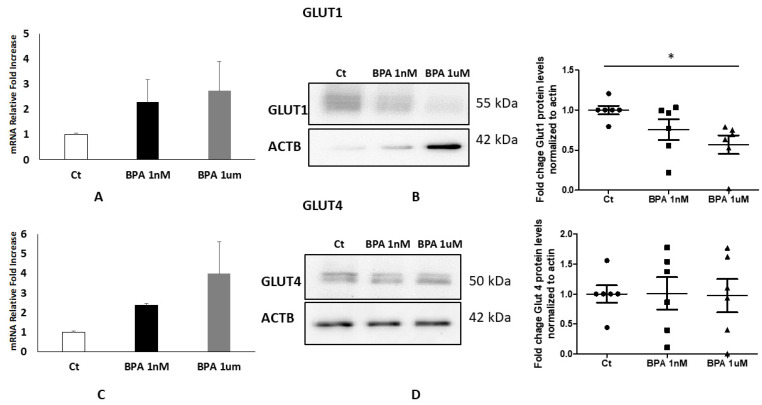
GLUT 1 and 4 expressions (**A**,**C**) and protein levels (**B**,**D**) in placenta explants treated with BPA from overweight women. (**A**) Fold Change of GLUT1 mRNA in placental explants from OW women (*n* = 5) treated with BPA 1 nM and BPA 1 μM compared to the vehicle as control. (**B**) Representative WB (left panel) and corresponding densitometry (right panel) of GLUT1 in placental explants from OW women (*n* = 6) treated with BPA 1 nM and BPA 1 μM compared to the vehicle as control. (**C**) Fold Change of GLUT4 mRNA in placental explants from OW women (*n* = 5) treated with BPA 1 nM and BPA 1 μM compared to the vehicle as control. (**D**) Representative WB (left panel) and corresponding densitometry (right panel) of GLUT4 in placental explants from OW women (*n* = 6) treated with BPA 1 nM and BPA 1 μM compared to the vehicle as control. Data are presented as mean ±ES. Significance was determined using a one-way ANOVA and Bonferroni’s test for post hoc comparisons * *p* < 0.05. Ct: control.

The placenta from women with a high BMI showed an altered metabolism [17]. Therefore, to examine whether the expression of the glucose transporters in high BMI placentae could be modified by exposure to BPA, placental explants from overweight women were treated with different doses of BPA or with the vehicle as a control. As shown in Figure 4a,c, GLUT1 and GLUT4 RNA messengers did not reveal any significant changes after the treatments. Nevertheless, a Western blot and relative densitometry (Figure 4b) showed a dose-response reduction in GLUT1 levels in BPA-treated explants compared to controls, statistically significant and in particular a significant decrease at 1 μM of treatment (*p* < 0.05-Fold Change 0.5681). As we observed for the explant from normal weight women, the one from overweight pregnancy also did not demonstrate any evident alteration in the GLUT4 gene and protein expression.

## 3. Discussion

In the present study, we demonstrated that, while no differences in GLUTs expression were detected between normal weight (NW) and overweight (OW) placentae, differences were observed in cultured placental explants and in response to BPA. In particular, GLUT1 was increased in BPA-treated NW villous explants and decreased in OW ones. GLUT4 was lower in OW than in NW villous explants and was not regulated by BPA.

The placenta is the fundamental organ in maintaining pregnancy and assuring fetal development and growth. While the placenta has adapted, over the millennia, to protect the fetus from environmental threats (infections and many other pitfalls such as glaciations or arid environments), it is defenseless against chemicals that are increasingly produced by humans. Thus, the fetus is inevitably exposed to many of the chemicals that come from the environment and pass through the placenta, such as BPA. The placenta can also be adversely affected by these substances. Specifically, BPA alters the secretion of human chorionic gonadotropin (hCG) and reduces cell migration and invasion of the human trophoblast [18,19]. According to an in vivo study conducted on pregnant mice, exposure to low-dose BPA was associated with developing features similar to preeclampsia, including hypertension, altered levels of sFlt-1/PIGF ratio, and kidney damage [20].

A pregnant mother may have been exposed to BPA for long periods of her life and suffer from the effects of this chemical, including metabolic dysfunctions [21]. These effects may affect fetal growth, as demonstrated by the high incidence of macrosomic fetuses in obese mothers [22]. A cohort study carried out in Korea on 788 couples (mother-son) demonstrated that exposure to BPA is negatively linked with intrauterine linear growth and positively correlated with volume growth during childhood [23]. Recent evidence showed that low doses of BPA (50 μg/kg/day) administered to pregnant mice during the first week of gestation induced the abnormal remodeling of the maternal spiral arteries resulting in IUGR [24].

According to the Developmental Origins of Health and Disease (DOhad), maternal diet, environmental insults, and lifestyle can interfere with the fetus-placental development, thus setting the conditions for future diseases later in life [25]. In this study, we investigated whether a high body max index and exposure to BPA could alter human placental function.

Glucose is the main source of nutrients and energy for the fetus and since the fetus is not able to carry out the gluconeogenesis process, the sugar is transported from the maternal to the fetal blood thanks to specific transporters in the placental barrier [26,27]. Alterations in glucose transport from the mother to the fetus could, therefore, have important and harmful consequences for the health of the offspring. The present work provided evidence that BPA can alter the expression of glucose transporters and that its effect is modulated by maternal body mass index during pregnancy.

Among the characterized glucose transporters, GLUT1 and GLUT4 are the most important in regulating glucose exchange at the placental level. GLUT1 is considered the primary placental glucose transporter [28]. Its expression increases during pregnancy [29] and is positively related to glucose intake [26]. GLUT1 is predominantly expressed by the syncytiotrophoblast (ST), the transporting epithelium of the placenta, with three folds higher density in the maternal-facing microvillous membrane (MVM) compared to the fetal-facing basal membrane (BM) [30,31]. The asymmetry in GLUT1 expression has indicated the higher glucose transport activity in the MVM, while the transport across the BM is the rate-limiting step of the transplacental glucose transfer [32]. GLUT4 is an insulin-dependent carrier. It is located in the ST cytoplasm and moved into the membrane following insulin stimulation. GLUT4 is expressed mainly during the first trimester of pregnancy and has a key role in maintaining glucose homeostasis in the early stages of fetal development [15]. Recent evidence revealed that insulin stimulation enhanced glucose uptake in the first trimester, but not in the term placenta, confirming that the insulin receptor and GLUT4 are markedly reduced in the third trimester of gestation. Therefore, in the term placenta, the glucose transfer occurs mainly through GLUT1 [15].

Pre-pregnancy BMI is highly correlated with GDM onset [33,34,35]. Current evidence indicates that GDM placentae are characterized by altered GLUT-1, GLUT-4, and GLUT-9 expression [36,37]. A recent meta-analysis showed a linear relationship between the risk of GDM and pre-pregnancy maternal BMI [38]. Accordingly, our data demonstrated that GDM developed in a higher percentage of OW patients with a higher pre-pregnancy BMI relative to CTRLs. Previous research linked lower gestational weight gain (GWG) with obesity’s severity showing that GWG declines as BMI increases [39]. Accordingly, we reported a lower GWG in OW women relative to CTRL. It is well established that obesity and GDM are associated with LGA neonates [40]. Moreover, it was described that LGA prevalence increased with increasing pre-pregnancy BMI among women with and without GDM [41]. We reported that OW women characterized by a higher pre-pregnancy BMI and by a higher percentage of GDM had a significant increase in LGA percentage relative to CTRL.

The GDM patients included in the present study were well controlled by diet and, even if a significant increase in glycemic levels was detected during the third trimester in OW compared to NW women, they were within physiological ranges.

Inconsistent results exist for GLUT expression and activity in GDM patients probably due to differences in criteria for GDM diagnosis as well as the complexity of the disease and the several factors that could impact placental function [42]. For these reasons and the fact that glycemia was physiological, we have grouped and studied the overweight patients all together for GLUT levels. No significant alterations of GLUT1 and 4 were noticed in OW placentae compared to the NW ones. According to Barros et al. [30], to focus on the expression and protein levels of the GLUTs in the trophoblast layer, we performed in vitro cultures of placenta explants obtained by NW and OW women. Our data indicated an increase, although not significant, in the expression of GLUT1 in placentae from OW women compared to the normal one and agreed with the studies of Acosta et al. [43]. The authors observed that GLUT1 levels raised in the basal membrane and microvillous membrane of the placentae from obese compared to normal weight women. We reported higher triglycerides levels in OW pregnant women during the early second and third trimesters. In rat placenta obtained from animals fed on a high-fat diet, the GLUT1 protein levels increased up to five times compared to controls [44]. Therefore, we hypothesized that the increased GLUT1 levels in the OW group could be connected to the high-fat diet. Moreover, GLUT4 expression and protein levels decreased significantly in the placental explants of OW women compared to NW women confirming previous results by Colomiere and James-Allan [45,46].

As previously described, BPA is an “obesogenic” factor. We, therefore, studied the expression of glucose transporters in BPA treated villous explants from NW and OW women. First of all, we observed, in explants obtained from NW women, an increase in GLUT1 expression as a result of exposure to BPA. The increase in GLUT1 levels was observed at low doses of the endocrine disrupter (environmentally relevant dose), confirming data by Benincasa et al. and Rajakumar et al. [14,47]. On the other hand, we detected a dose-dependent decrease in the expression of GLUT1 in placenta explants from OW women after exposure to the endocrine disrupter. The impact of BPA was specific for GLUT1 since no significant effects were detected at any concentration on GLUT 4 in OW and NW placentae.

While supporting previous data on the effect of BPA in upregulating placental expression of GLUT1, our study shows for the first time to our knowledge that the placental response to BPA in mothers with metabolic dysfunction results in a reduction rather than an increase in GLUT1 physiological levels. Given the importance of glucose as a major source of nutrients and energy for the fetus, a worsening of its transport across the placenta as it occurs in overweight mothers can be detrimental to the fetal growth and development. On this basis, a maternal condition of overweight/obesity appears to be a risk factor for the protection of the fetus from contamination by environmental chemicals such as BPA.

There is an increasing number of overweight/obese women of reproductive age. Maternal obesity is affecting the continuation of pregnancy, premature birth, abortion, congenital abnormalities, fetal macrosomia, and a higher incidence of cardiovascular metabolic disorders in their offspring. The obese maternal environment is characterized by hyperlipidemia and an excessive state of inflammation as well as oxidative stress compared to a pregnant woman of normal weight [17]. Moreover, it has been reported that the nutritional status of the mother can alter the epigenetic state and, as a consequence, the gene expression of the placental and fetal genome [48]. More recent studies have confirmed an alteration in the placental methylome of obese compared to normal weight women [49]. Therefore, we hypothesize that the modified placental epigenetic profile from overweight women may lead to altered GLUT1 expression after exposure to BPA.

Further studies will focus on elucidating the mechanisms and pathways involved in the different placental responses between OW and NW mothers. It will also be important to examine the different impacts of BPA on glucose transport in large or small for gestational age fetuses.

## 4. Materials and Methods

### 4.1. Ethics Statement and Place of Recruitment

This study was conducted according to the principles expressed in the Declaration of Helsinki. The study was approved by the Institutional Review Board of O.I.R.M. S. Anna Hospital and “Ordine Mauriziano di Torino” (n.CS2/297; protocol n 0061542; 21/06/2017) (Turin, Italy). All patients were recruited at O.I.R.M S. Anna Hospital (Turin, Italy) and provided written informed consent for the collection of samples and subsequent analysis.

### 4.2. Study Population, Definitions and Tissues Collection

From June 2017 to June 2020, 39 overweight women (OW) and 44 age-matched normal weight women (NW, Controls-CTRL) with singleton spontaneous pregnancies were recruited and interviewed before 14 weeks of gestation. OW (*n* = 13) and NW (*n* = 12) dropouts were excluded, leaving 26 OW and 39 NW pregnant women for the statistical analysis. Before 14 weeks of gestation and after 27 weeks of gestation, maternal venous blood samples (5 mL) were collected into Vacutainer tubes with and without anticoagulant. Serum and plasma were separated by centrifugation (3000× *g* rpm at 4 °C for 20 min) and stored at −20 °C until assayed. For each woman, hemochrome, standard coagulation, glycemia, triglycerides, total cholesterol, High-Density Lipoprotein Cholesterol (HDL-C), Thyroid-Stimulating Hormone (TSH), Free Thyroid Hormones 4 (FT4), vitamin D, and homocysteine tests were measured using an autonomic analyzer.

The evaluation of the uterine arteries took into account resistance index (RI) or Pourcelot ratio, defined as peak systolic flow minus peak end diastolic flow divided by peak systolic flow. According to the literature, an abnormal uterine artery Doppler FVW was defined as a mean (of the two uterine arteries) RI of ≥0.58; a further, more restrictive cut-point (≥0.62) was also tested. Both cut-points are standardized references and are applied to gestational ages of at least 26 weeks. The uterine flows are not physiologically variable after the gestational age, and not age-adjusted [45]. Umbilical artery Doppler waveforms were analyzed using the Pulsatility index (PI), defined as peak systolic flow minus end diastolic flow divided by mean flow. Normal values of PI were adjusted for gestational age, after the 26th gestational week, and were defined according to gestational age-adjusted data proposed by Todros et al. [50].

Overweight and Obesity were diagnosed in categories of body mass index (BMI) as defined by the World Health Organization: BMI < 18.5 kg/m^2^: underweight; BMI 18.5–24.9 kg/m^2^: normal weight; BMI 25.0–29.9 kg/m^2^: overweight; BMI 30.0–34.9 kg/m^2^: class I obesity; BMI 35.0–39.9 kg/m^2^: class II obesity; and BMI ≥ 40.0 kg/m^2^: class III obesity [9,51].

GDM diagnosis was performed as previously described by Nuzzo et al. [52]. Briefly, the oral glucose tolerance test (OGTT) was performed by administrating 75 g of glucose between 16 and 18 weeks of gestation in women with at least one high-risk factor. In the case of normal OGTT results, the test was repeated at 24–28 weeks of gestation. Women with one or more plasma glucose values above the established thresholds (≥92 mg/dL at baseline, ≥180 mg/dL after 1 h from load, ≥153 mg/Dl after 2 h from load) were diagnosed as GDM [52]. In our cohort, all the GDM patients routinely received dietary counseling and nutritional recommendations in line with guidelines [53]. Furthermore, 30 min daily moderate exercise was recommended (i.e., brisk walking).

According to the Italian neonatal anthropometric reference charts [54], newborns were classified as Large for Gestational Age (LGA) when birth weight and/or length and/or abdominal circumference (AC) >90th percentile.

Pregnancies with congenital malformations, chromosomal disorders (in number or structure), evident intra-uterine infections, as well as patients with diabetes, infections, chronic hypertension, kidney disease, or smoke and/or alcohol abuse were excluded.

### 4.3. Human Chorionic Villous Explants Cultures and BPA Treatment

Biopsies from normal weight (*n* = 6) and overweight placentae (*n* = 6) were processed. Amniotic membranes were mechanically removed, and placental specimens were washed in cold phosphate-buffered saline (PBS) solution to eliminate excess blood. Explants cultures were then performed as described by Caniggia et al. [55] with some modifications. Briefly, small portions of placental chorionic villi (5–10 mg wet weight) were excised and placed in 96-well culture dishes. Explants were cultured in DMEM F12 (phenol red and serum free) plus L-glutamine (Gibco, Invitrogen, Basel, Switzerland) and 1% antibiotics (penicillin-streptomycin) (Sigma Chemical Co., St. Louis, MO, USA) and incubated at 37 °C and 5% CO_2_ to equilibrate overnight. Explants were then removed from the culture media and placed in 200 μL of medium with different BPA concentrations: 1 nM and 1 μM. Explants in basal culture medium were used as controls. Finally, control and treated explants were collected after 48 h and immediately frozen for RNA and protein isolation. The supernatants were centrifuged and used for lactate dehydrogenase activity (LDH). This model allowed us to determine the sequence of molecular events in tissues characterized by conserved physiological pathways, thus avoiding biases due to previous existing pathological anomalies.

### 4.4. RNA Isolation and Real Time PCR

Total RNA was isolated from placental biopsies and villous explants using a TRIzol reagent (Life Technologies, Invitrogen, Carlsbad, CA, USA, Cat. No. t9424) according to the manufacturer instructions. Genomic DNA contamination was removed by DNase I digestion before RT-PCR. RNA was quantified using a NanoDrop Microvolume Spectrophotometer (ThermoFisher Scientific, Waltham, MA, USA), and cDNA was generated from one µg of total RNA using a random hexamers approach and RevertAid H Minus First Strand cDNA Synthesis kit (Fermentas, Waltham, MA, USA, Cat.No k1632). qRT-PCR reactions were run in the StepOne™ Real-Time PCR System instrument (Applied Biosystems Group, Waltham, MA, USA). Gene expression levels of GLUT1 (Life Technologies, Cat. No 4331182, seq. Hs00892681_m1) and GLUT4 (Life Technologies, Carlsbad, MA, USA, Cat. No 4331182, seq. Hs00168966_m1) were determined by Real-Time PCR using specific TaqMan commercially available inventoried primers and probes. mRNA levels were normalized using endogenous 18S (Life Technologies, Cat. No 4333760F) and GAPDH (Glyceraldehyde 3-phosphate dehydrogenase), (Life Technologies, Cat. No 4331182, seq. Hs02786624_g1) as internal references. The expression level of the selected genes was calculated by the 2^−ΔΔCt^  [56].

### 4.5. Western Blot Analyses

Total proteins from placental biopsies or chorionic villous explants were homogenized in RIPA buffer (50 mM Tris-HCl, pH 7.5; 150 mM NaCl; 1% (*vol/vol*) Triton X-100; 1% (*wt/vol*) sodium deoxycholate; 0.1% (*wt/vol*) SDS; 1 mM Na_2_VO_3_; 25 mM NaF; and protease inhibitors cocktail (Sigma Chemical Co., St. Louis, MO, USA) and centrifuged at 15,000× *g* at 4 °C for 15 min. Protein concentrations of the supernatants were then determined using Bradford protein assay (Biorad Laboratories, Milan, Italy).

Western blot (WB) analyses were performed as previously described [57]. Briefly, 30 or 60 μg of total protein lysates obtained respectively from villous explants and placental samples were separated in 10% SDS-PAGE gel and electro-transferred onto PVDF membranes. Blots were then saturated in Tris-buffered saline pH 7.2 (TBS) containing 4% non-fat dry milk and 0.1% Tween 20 for 1 h at room temperature. After that, membranes were incubated overnight at 4 °C with primary antibodies. Blots were then washed and incubated for 60 min at room temperature with the appropriated horseradish peroxidase-conjugated secondary antibodies. The reaction was revealed using a chemiluminescent reagent (BioRad Laboratories Inc., Cambridge, MA, USA) and membranes digitalized with CHEMI DOC (BioRad Laboratories Inc.). Densitometric analysis of WB was performed using ImageJ (NIH). Samples were normalized to beta Actin.

### 4.6. Antibodies Used in the Research

Primary antibodies employed in Western blotting include mouse monoclonal anti-beta Actin (ACTB) (sc-81178, 1:1000, Santa Cruz), mouse monoclonal anti-GLUT4 (2213; 1:1000, Cell Signaling), rabbit polyclonal anti-GLUT1 (PA5-16793; 1:1000, Thermo Fisher Scientific^®^). HRP-conjugated secondary antibodies were obtained from BioRad (BioRad Laboratories Inc.) and used at a concentration of 1:3000.

### 4.7. Statistical Analyses

Data are represented as mean ± ES for parametric and as median and range for non-parametric data. Comparison among groups was performed by analysis of variance. Bonferroni’s test was used for post hoc comparisons between two groups of parametric data, while Kruskal–Wallis test was used for non-parametric data. When two groups were compared, an Unpaired Student’s *t*-test or Mann-Whitney test was used. Categorical variables are presented as frequencies (percentages), and the comparison between different groups was made with a Chi-Square Test. Statistical tests were carried out using SPSS Version 27 statistical software or GraphPad Prism 7.0 software (San Diego, CA, USA). Significance was accepted at *p* < 0.05.

## Figures and Tables

**Table 1:** Maternal pre-pregnancy characteristics and outcomes in normal weight and overweight pregnancies. Values are expressed as mean ± ES and percentage. Significant differences (*p* < 0.05): * differences indicating a significant effect compared with normal weight. *t*-test and chi-square test were used.

	Normal Weight (*n* = 32)	Overweight (*n* = 26)	*p* Value
**Pre-Pregnancy**			
**Nulliparae (%)**	46.9	22.2 *	*p* = 0.049
**Maternal age (years)**	33.4 ± 0.6 (25–39)	33 ± 0.7 (24–39)	*p* > 0.05
**Caucasian ethnicity (%)**	100	100	*p* > 0.05
**Pre-pregnancy weight (Kg)**	59 ± 1.3	80.3 ± 1.9 *	*p* < 0.001
**Height (cm)**	166 ± 1.3	163.6 ± 1.1	*p* > 0.05
**Pre-pregnancy BMI (kg/m^2^)**	21.4 ± 0.3	30 ± 0.5 *	*p* < 0.001
**Outcomes**			
**Delivery BMI (kg/m^2^)**	26.4 ± 0.4	33.5 ± 0.6 *	*p* < 0.001
**GWG (kg)**	13.8 ± 0.7	9.4 ± 0.9 *	*p* < 0.001
**Plicometry (cm)**	2.5 ± 0.1	3.3 ± 0.1 *	*p* < 0.001
**Mid-upper arm circumference (cm)**	25.2 ± 0.4	30.2 ± 0.7 *	*p* < 0.001
**Systolic Blood Pressure (mm Hg)**	114.7 ± 2.3	122.3 ± 1.5 *	*p* = 0.012
**Diastolic Blood Pressure (mm Hg)**	71.6 ± 1.4	72.3 ± 1.6	*p* > 0.05
**GDM (%)**	12.5	37 *	*p* = 0.027
**GDM Therapy (%)**	Diet: 100 Insulin: 0	Diet: 100 Insulin: 0	*p* > 0.05

**Table 2:** Maternal hematological parameters in normal weight and overweight women in early second trimester and third trimester pregnancies. Values are expressed as mean ± SE and percentage. Significant differences (*p* < 0.05): ^ differences indicating a significant effect compared with NORMAL WEIGHT 2tr; ° differences indicating a significant effect compared with Homologous Overweight 2tr; ^§^ differences indicating a significant effect compared with Homologous Normal Weight 3tr. One-way ANOVA with Bonferroni’s post hoc test was used.

	NORMAL WEIGHT ≤ 14 Weeks (*n* = 32)	OVERWEIGHT ≤ 14 Weeks (*n* = 26)	NORMAL WEIGHT ≥ 27 Weeks (*n* = 32)	OVERWEIGHT ≥ 27 Weeks (*n* = 26)	*p* Value
**Gestational age at blood sampling (weeks)**	13.4 ± 0.2	13.2 ± 0.2	35 ± 0.4	34.4 ± 0.5	*p* > 0.05
**Maternal blood parameters:**					
**Glycemia (mg/dL)**	72.8 ± 1.2	76.8 ± 2.2	68.9 ± 2.1	79.2 ± 2.3 ^§^	NW 3tr vs. OW 3tr: *p* = 0.006
**Triglycerides (mg/dL)**	104.5 ± 7.4	154.7 ± 20.5 ^^^	214.1 ± 16.4 ^^^	254.3 ± 23.1 ^°^	NW 2tr vs. OW 2tr: *p* = 0.018 NW 2tr vs. NW 3tr: *p* < 0.001 OW 2tr vs. OW 3tr: *p* = 0.002
**Total cholesterol (mg/dL)**	195.2 ± 6.8	199.9 ± 5.2	259.6 ± 10.3	247.3 ± 8.1°	OW 2tr vs. OW 3tr: *p* < 0.001
**HDL cholesterol (mg/dL)**	78.5 ± 2.5	71.4 ± 2.3	75.7 ± 3.4	76.5 ± 3	*p* > 0.05
**TSH (mU/L)**	1.7 ± 0.2	1.6 ± 0.2	1.8 ± 0.2	1.8 ± 0.2	*p* > 0.05
**FT4 (ng/dL)**	8.2 ± 1	7.2 ± 1	6.7 ± 1	7.1 ± 0.9	*p* > 0.05
**Vit. D (ng/mL)**	28.6 ± 6	33.7 ± 7.9	51.5 ± 14	43 ± 10.1	*p* > 0.05
**Homocysteine (mcmol/L)**	6.1 ± 0.2	6.3 ± 0.2	6.2 ± 0.3	6.8 ± 0.7	*p* > 0.05
**White Bood Cells (10^9^/L)**	7.95 ± 0.3	8.6 ± 0.5	9.3 ± 0.3	9.9 ± 0.5	*p* > 0.05
**Red Blood Cells (10^12^/L)**	4.1 ± 0.1	4.1 ± 0.01	3.9 ± 0.1	4.15 ± 0.1	*p* > 0.05
**Hemoglobin** **(g/dL)**	12.2 ± 0.2	12.6 ± 0.2	11.3 ± 0.2 ^^^	11.8 ± 0.2	NW 2tr vs. NW 3tr: *p* = 0.013
**Platelet** **(10^9^/L)**	238 ± 12.5	248 ± 9.8	222.4 ± 13.4	247.1 ± 13.4	*p* > 0.05

**Table 3:** Fetal outcomes in normal weight and overweight pregnancies. Values are expressed as mean ± ES and percentage. Significant differences (*p* < 0.05): * differences indicating a significant effect compared with normal weight. *t*-test and chi-square test were used.

	Normal Weight (*n* = 32)	Overweight (*n* = 26)	*p* Value
**Gestational age at delivery (weeks)**	39.9 ± 0.2	39.6 ± 0.2	*p* > 0.05
**Caesarian section (%)**	31.2	22.2	*p* > 0.05
**Placental weight (g)**	581.6 ± 17.4	584.5 ± 12.9	*p* > 0.05
**Birth weight (g)**	3493.1 ± 58.8	3401.8 ± 71.4	*p* > 0.05
**Fetal to placenta weight ratio**	6.1 ± 0.1	5.9 ± 0.1	*p* > 0.05
**Sex of infant**			
**Female (%)**	37.5	40.7	*p* > 0.05
**Male (%)**	62.5	59.3	*p* > 0.05
**Large for Gestational Age, AC > 90 centile (%)**	3.1	22.2 *	*p* = 0.024
**5-min Apgar score ≤7 (%)**	0	0	*p* > 0.05
**NICU (%)**	0	0	*p* > 0.05

## Data Availability

The raw data supporting the conclusions of this article will be made available by the authors, without undue reservation.
